# All-optical regenerator of multi-channel signals

**DOI:** 10.1038/s41467-017-00874-0

**Published:** 2017-10-12

**Authors:** Lu Li, Pallavi G. Patki, Young B. Kwon, Veronika Stelmakh, Brandon D. Campbell, Muthiah Annamalai, Taras I. Lakoba, Michael Vasilyev

**Affiliations:** 1 Department of Electrical Engineering, University of Texas at Arlington, Arlington, TX 76019 USA; 2 Department of Mathematics and Statistics, University of Vermont, Burlington, VT 05401 USA; 3 Present Address: TE SubCom, Eatontown, NJ 07724 USA; 4 Present Address: Infinera Corp., Bangalore, 560025 India; 5 Present Address: Lightwave Logic, Inc., Longmont, CO 80501 USA; 6 Present Address: Department of Electrical Engineering and Computer Science, Massachusetts Institute of Technology, Cambridge, MA 02139 USA; 7 Present Address: Synopsys, Inc., Mountain View, CA 94043 USA

## Abstract

One of the main reasons why nonlinear-optical signal processing (regeneration, logic, etc.) has not yet become a practical alternative to electronic processing is that the all-optical elements with nonlinear input–output relationship have remained inherently single-channel devices (just like their electronic counterparts) and, hence, cannot fully utilise the parallel processing potential of optical fibres and amplifiers. The nonlinear input–output transfer function requires strong optical nonlinearity, e.g. self-phase modulation, which, for fundamental reasons, is always accompanied by cross-phase modulation and four-wave mixing. In processing multiple wavelength-division-multiplexing channels, large cross-phase modulation and four-wave mixing crosstalks among the channels destroy signal quality. Here we describe a solution to this problem: an optical signal processor employing a group-delay-managed nonlinear medium where strong self-phase modulation is achieved without such nonlinear crosstalk. We demonstrate, for the first time to our knowledge, simultaneous all-optical regeneration of up to 16 wavelength-division-multiplexing channels by one device. This multi-channel concept can be extended to other nonlinear-optical processing schemes.

## Abstract (web summary)

Nonlinear optical processing devices are not yet fully practical as they are single channel. Here the authors demonstrate all-optical regeneration of up to 16 channels by one device, employing a group-delay-managed nonlinear medium where strong self-phase modulation is achieved without nonlinear inter-channel crosstalk.

## Introduction

All-optical regeneration is a striking example of the need for wavelength-division-multiplexing (WDM) compatibility in nonlinear-optical signal processing. All-optical regenerators reset to their original shape the signals that have accumulated noise and distortion due to propagation in fibre communication lines. In the simplest case of 2R regenerators, re-amplification and re-shaping takes place. In 3R regenerators, a function of re-timing is added. Although all-optical regenerators can be data-rate independent and work with much higher (Terabits per second) maximum rates than their optoelectronic counterparts, the latter offer more advanced capabilities (e.g. forward error correction) and lower manufacturing cost. Thus, to be practically viable, one all-optical regenerator must be able to replace a large number of optoelectronic regenerators, which makes its compatibility with WDM the most important challenge^1, 2^. This challenge, however, is of fundamental nature, because the threshold-like input–output power transfer function of a 2R regenerator requires strong optical nonlinearity, which necessarily leads to cross-phase modulation (XPM) and four-wave mixing (FWM) interaction among the WDM channels. While the re-timing function of 3R regenerator can, at least in principle, be performed for all WDM channels by a single device^3–6^ (though under rather impractical assumptions of synchronised clock rates for all channels), the WDM operation of a 2R regenerator has remained a fundamental obstacle up until now.

The efforts on overcoming this problem have intensified over the last decade and can be roughly arranged into three approaches. The first approach is to use spatial degrees of freedom to isolate the WDM channels from interacting with one another, which means that the channels are de-multiplexed and subsequently processed by separate nonlinear media^4, 7–10^. Second approach is to employ a single nonlinear medium, but use other degrees of freedom, such as polarisation^11^ and opposite directions of propagation^12, 13^, to isolate WDM channels. This approach has led to the demonstration of simultaneous regeneration of four channels in the same nonlinear fibre^14^. The third approach is to employ very low spectral efficiency in WDM transmission, which can help minimise interaction among the WDM channels either by placing the channels far from each other in frequency domain, or by separating the bits far from each other in time domain (i.e. using low-duty-cycle pulses). For the time-domain-separation method, the low duty cycle suppresses the interaction by ensuring that neighbouring-channel pulses very rarely overlap in time^15, 16^. However, the low-duty-cycle pulses require bandwidth greatly exceeding their repetition frequency (and hence greatly exceeding their data rate), i.e. they are not spectrally efficient. Another variant of time-domain-separation method involves interleaving the pulses from different WDM channels in time^16–19^, but it requires bit synchronisation among the channels, which is not possible to achieve in real communication systems. For frequency-domain-spacing method, the interaction is suppressed by the presence of substantial chromatic dispersion of the fibre, which makes the neighbouring-channel pulse streams rapidly walk off from each other, thereby eliminating FWM and reducing XPM to merely a constant phase shift proportional to the average power of the neighbouring channel. However, the dispersion also causes the different frequency components of the data-carrying pulses within each WDM channel to walk off from each other. This distorts the pulse shape and eliminates the regenerative effect of the optical nonlinearity unless the ratio of the bandwidth of each WDM channel to the inter-channel spacing, known as spectral efficiency, is very small. Thus, none of the approaches discussed above have shown any potential for increasing the number of regenerated channels beyond four, unless the channels carry identical data and/or their bits are synchronised in time^16, 19, 20^, which is impractical.

A radically different method of WDM regeneration was proposed by us in ref. ^21^. This approach uses the benefits of the dispersive walk-off between the WDM channels to avoid FWM and XPM, but at the same time eliminates the walk-off among the frequency components within each channel to preserve pulse integrity and enable accumulation of large amounts of self-phase modulation (SPM). In order to achieve this, we proposed to make an artificial group-delay-managed (GDM) nonlinear medium consisting of a series of short nonlinear fibre sections separated by spectrally periodic phase filters known as periodic group-delay devices (PGDDs). The GDM medium has chromatic dispersion that is periodic both in propagation direction and in frequency, and is described in detail in the ‘Results’ section. While its unique properties can enable the WDM operation in any SPM-based 2R regenerator, for the purpose of demonstration we focus on the regenerator of on–off-keying (OOK) signals, based on SPM spectral broadening followed by spectral filtering (Mamyshev regenerator scheme^22^).

In this paper, we report the demonstration of the first, to our knowledge, truly multi-channel 2R regenerator, which uses the Mamyshev scheme enabled by the GDM nonlinear medium. The presented work consists of two parts: in the first one the regeneration with GDM medium is studied by placing one GDM unit cell consisting of a fibre section and a PGDD into a recirculating loop^23, 24^ and letting the signals propagate through this cell multiple times; in the second one we use the obtained insights to build the first stand-alone GDM-enabled Mamyshev regenerator and demonstrate simultaneous regeneration of 16 WDM channels in it. We have reported preliminary 3- and 12-channel stand-alone regeneration data in two recent conference papers^25, 26^. This paper presents the experiment with optimised dispersion map and Raman amplification, yielding the eye-opening improvements that are significantly greater and more equalised among the channels, and resulting in regeneration of the largest number (16) of WDM channels to date. (While this manuscript was under review, we learned of a more recent work reporting regeneration of 16 channels with 50-GHz spacing, albeit requiring bit synchronisation among all channels: Guan et al.^27^.) The GDM-based approach can be straightforwardly extended to higher channel counts and higher symbol rates, and we believe it can also be adapted for regeneration of more advanced modulation formats.

## Results

### Group-delay-managed nonlinear medium

**Fig. 1:**
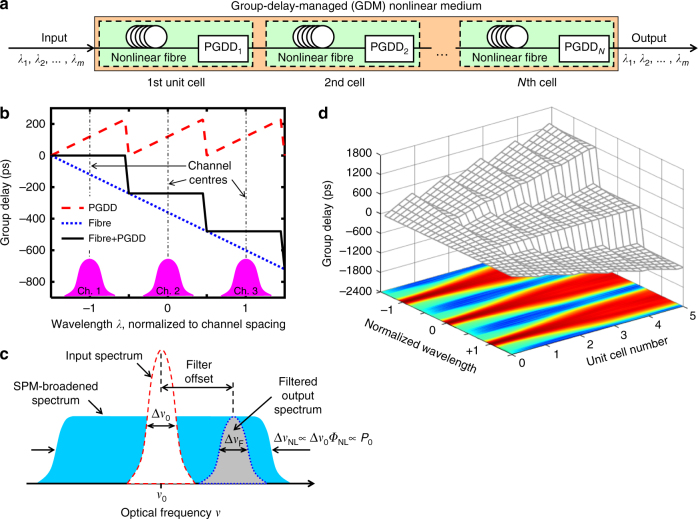
Principle of multi-channel 2R regeneration. **a** Group-delay-managed (GDM) medium is made by concatenation of *N* ʽfibre + periodic group-delay device (PGDD)ʼ unit cells and processes *m* WDM channels. **b** Each cell has a staircase-like group-delay spectrum (whose derivative is dispersion), ensuring strong nonlinearity within each channel, but suppressed nonlinear interactions among the channels. Group-delay spectra are shown for three adjacent WDM channels. **c** Single-channel Mamyshev 2R regenerator is based on signal’s spectral broadening by self-phase modulation (SPM), followed by off-centre bandpass filtering. **d** Propagation in the GDM medium results in group-delay walk-off between the adjacent channels (wavelength is normalised to channel spacing) and allows their simultaneous broadening by the SPM without nonlinear inter-channel crosstalk. The *coloured map* underneath represents evolution of power spectral densities of the three channels. Group delay values in **b**, **d** assume the channel spacing of 200 GHz (1.6 nm) and fibre with dispersion *D* = –120 ps nm^−1^ km^−1^ and section length of 1.25 km

Our approach, originally proposed in ref. ^21^, uses the dispersive walk-off between the WDM channels to avoid FWM and XPM, but at the same time produces no walk-off among the frequency components within each channel, which preserves pulse integrity and enables accumulation of large amounts of SPM. In order to achieve this, we split the nonlinear fibre used for 2R regeneration into a series of short sections separated by spectrally periodic phase filters known as PGDDs, as shown in Fig. 1a. Such filters, usually made of several cascaded Gires–Tournois etalons^28, 29^, have sawtooth-like group-delay spectra (Fig. 1b). When the PGDD spectrum is added to a straight-line (i.e. constant-dispersion) group-delay spectrum of a section of the nonlinear fibre, the resulting group-delay spectrum of the ʽfibre + PGDDʼ combination exhibits staircase-like behaviour (Fig. 1b). This ensures equal delays (i.e. no dispersion) among all frequency components within each channel, thereby maximising SPM, but at the same time introduces temporal walk-off between different WDM channels, which reduces XPM and FWM. When multiple ʽfibre + PGDDʼ unit cells are concatenated, such walk-off is accumulated, effectively resulting in the creation of a new artificial nonlinear medium with large group-velocity dispersion among different WDM channels and with no dispersion within each channel. In such a medium, which we refer to as ʽGDM nonlinear medium,ʼ the benefits of large SPM can be enjoyed simultaneously by each of many WDM channels without suffering from the FWM and XPM. In this paper we focus on a GDM medium based on nonlinear fibres and commercially available PGDDs^29, 30^. In the future, it might be possible to implement the entire GDM on a chip using integrated-photonics solutions for both nonlinear^31, 32^ and PGDD^33–35^ functions.

The use of bit walk-off to suppress XPM was originally proposed for conventional long-haul communication lines^36, 37^ and later was demonstrated with PGDDs for dispersion-managed soliton transmission^38, 39^. The long-haul communication lines, however, operate in the regime where the nonlinearities are weak, whereas 2R regeneration inherently relies on large SPM, i.e. operates in a strongly nonlinear regime. We found the optimum parameters of operation for the latter regime^40^, which indicate that an excellent 2R performance, which is also very robust with respect to perturbations of the experimental parameters, can be obtained with as few as five or six ʽfibre + PGDDʼ unit cells with anomalous net dispersion of the cell and large normal dispersion of the nonlinear fibre.

The GDM nonlinear medium can enable the WDM operation in any SPM-based 2R regenerator or other optical signal processor. In this paper we report its application to a particular scheme known as the Mamyshev regenerator^22^. The operation of the latter is illustrated in Fig. 1c for the case of a single channel. After propagation in nonlinear fibre, a noisy input pulse (ʽONEʼ symbol) with original bandwidth Δ*ν*_0_ experiences SPM-caused spectral broadening so that the width of the broadened spectrum Δ*ν*_NL_ is proportional to the input pulse’s peak power *P*_0_ while the power spectral density of the broadened spectrum is virtually independent of the input power. Hence, by selecting a portion of the broadened spectrum by an optical bandpass filter (OBPF), one can obtain an output pulse of approximately the same duration as the input pulse (if OBPF width Δ*ν*_F_ ≈ Δ*ν*_0_), but with the magnitude that does not change with input power fluctuations (regeneration of ʽONESʼ symbols). On the other hand, any noise between the pulses (i.e. taking place of ʽZEROʼ symbols) is too weak to cause SPM broadening and is confined within the input signal’s bandwidth. If the OBPF centre frequency is offset from the centre of the input signal’s spectrum, the noise between the pulses is not transmitted to filter’s output, which constitutes the regeneration of ʽZEROʼ symbols. For proper operation, the Mamyshev scheme requires large amount of SPM (3…12 radians of nonlinear phase shift Φ_NL_). Figure 1d illustrates application of the GDM medium to the Mamyshev regenerator.

The compatibility of the Mamyshev regenerator with conventional dispersion management has been experimentally demonstrated for a single channel^41^ and later investigated with three to four channels^15, 42^. These experiments used alternating normal- and anomalous-dispersion fibres and did not involve PGDDs. Such conventional dispersion management schemes do not monotonically accumulate walk-off between channels and, hence, are unable to sufficiently suppress XPM and FWM. As a result, these experiments could not be extended to multi-channel operation.

In this paper, we demonstrate multi-channel 2R regeneration in the Mamyshev scheme enabled by the GDM nonlinear medium. In all presented experiments the nonlinear fibre is the conventional dispersion-compensating fibre (DCF) with nonlinear constant *γ* ≈ 5 (W km)^−1^, dispersion *D = *–120 ps nm^−1^ km^−1^, and attenuation ≈ 0.5 dB km^−1^. All signals are OOK modulated by a 2^31^–1 pseudo-random bit sequence (PRBS) and carved into 50% return-to-zero (RZ) pulses. To ensure the worst case of inter-channel nonlinearities (XPM and FWM), all WDM signals are co-polarised. In order to properly characterise the 2R regeneration performance, we decorrelate the clock frequencies and the bit patterns between the neighbouring channels (see ‘Methods’ for more details). Without proper clock decorrelation, one is likely to observe unrealistically optimistic regeneration performance (e.g. when the 50% RZ pulses from the neighbouring channels are interleaved in time), which cannot be achieved in a practical communications system, where the channels have independent (uncorrelated) clocks.

### Multi-channel regeneration in a loop-based GDM medium

**Fig. 2:**
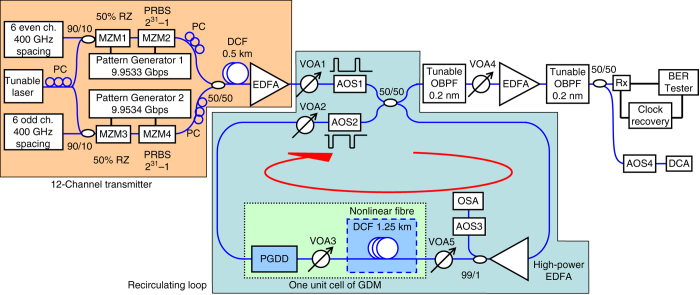
Experimental recirculating-loop setup. 10-Gbps-modulated signals from 12-channel transmitter enter the recirculating loop containing one GDM unit cell. After five circulations the signals are filtered and detected by a pre-amplified receiver (Rx). The tunable laser is used to generate ±25% amplitude jitter when it is tuned to the wavelength of the signal channel to be measured. MZM: electro-optic Mach–Zehnder modulator, EDFA: erbium-doped fibre amplifier, PC: polarisation controller, AOS: acousto-optic switch, VOA: variable optical attenuator, DCF: dispersion-compensating fibre, OSA: optical spectrum analyser, OBPF: optical bandpass filter, DCA: digital communication analyser

**Fig. 3:**
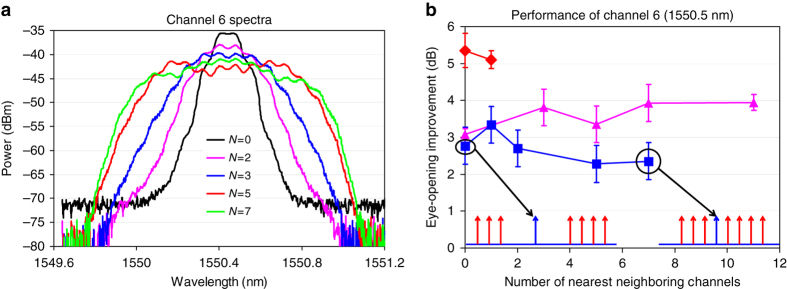
Multi-channel 2R regeneration in the recirculating loop. **a** SPM-broadened spectra of the central (1550.5 nm) channel (out of 12 channels total) after *N* circulations, obtained with the resolution bandwidth of 0.1 nm. **b** Eye-opening improvement at BER = 10^−9^ for the central 1550.5-nm channel after *N* = 5 circulations versus the number of other simultaneously regenerated channels. Different-coloured traces in **b** describe three separate experiments using different EDFAs in the loop and should not be compared to one another. *Red diamonds* correspond to the increase from 1 to 2 and the *purple triangles*—to the increase from 1 to 12 in the total number of consecutive 200-GHz-spaced WDM channels. *Blue squares* correspond to the change in the number of the central channel’s closest neighbours on the 200-GHz-spaced grid from 0 to 7, while the total number of channels is kept at 8. *Error bars* are obtained from the linear fits to BER curves (*red diamonds*, first and last *purple triangles*) and from power measurement uncertainties (*blue squares*, three middle *purple triangles*)

In order to be able to easily vary the number of ʽfibre + PGDDʼ unit cells in the regenerator without drastic increase in the required resources, and to experimentally confirm the parameters of the theoretically predicted regime^40^ of multi-channel regeneration in the GDM medium, we have built a recirculating loop^23, 24^, where we used only one ʽfibre + PGDDʼ unit cell and passed the signals through it multiple times (Fig. 2) to achieve the effect of concatenating multiple identical cells. After each pass (circulation), the spectrum of each WDM channel is increasingly broadened by SPM, until it completely fills the passband width of the PGDD’s amplitude response after five circulations (the PGDD in this experiment has a periodic bandpass amplitude characteristic with –1-dB width of 100 GHz), as shown in Fig. 3a. Hence, using a 10 Gigabit-per-second (Gbps) signal after five circulations as the regenerated output, we have studied the regeneration performance with various numbers of channels (Fig. 3b). Although 2R regeneration cannot improve the intrinsic bit-error rate (BER) of the signal, the compression of noise of ʽZEROʼ and ʽONEʼ symbols makes the signal more robust with respect to subsequent noise addition. This improvement (a.k.a. ʽeye-opening improvement,ʼ because it increases the opening between the ʽZEROʼ and ʽONEʼ levels on the eye diagram) is quantified by the reduction of the signal power level at the receiver’s optical pre-amplifier entrance that is required to obtain BER = 10^−9^. While the merits of a regenerator are ultimately determined by the reach extension it offers to a specific real transmission system in the field, the eye-opening improvement is the widely accepted laboratory measure of the regenerator’s ability to suppress the signal’s impairments and hence improve the system performance. In three separate experiments, we have found that the eye-opening improvement of the central channel degraded by amplitude jitter does not show any noticeable degradation when the number of WDM channels on a 200-GHz-spaced grid is increased from 1 to 2 (*red diamonds* in Fig. 3b), from 1 to 12 (*purple triangles*), or when the number of the channel’s closest neighbours on this grid is changed from 0 to 7 while keeping the total number of channels at 8 (*blue squares*). In general, the negative impact of XPM and FWM from neighbouring channels on the performance of a given regenerated channel quickly diminishes with the increased frequency separation between the channels. Thus, only a few (5–7) closest neighbours have a potential to degrade the signal channel’s regeneration performance. Our GDM method strongly suppresses this degradation: as the results in Fig. 3b show, the performance is not degraded as we change the number of closest neighbour channels from 0 to 11. Thus, in line with our theoretical prediction in ref. ^21^, these results experimentally demonstrate that there is no fundamental XPM or FWM limit on the number of channels regenerated in our scheme, i.e. adding more channels would not increase XPM or FWM impairments.

**Fig. 4:**
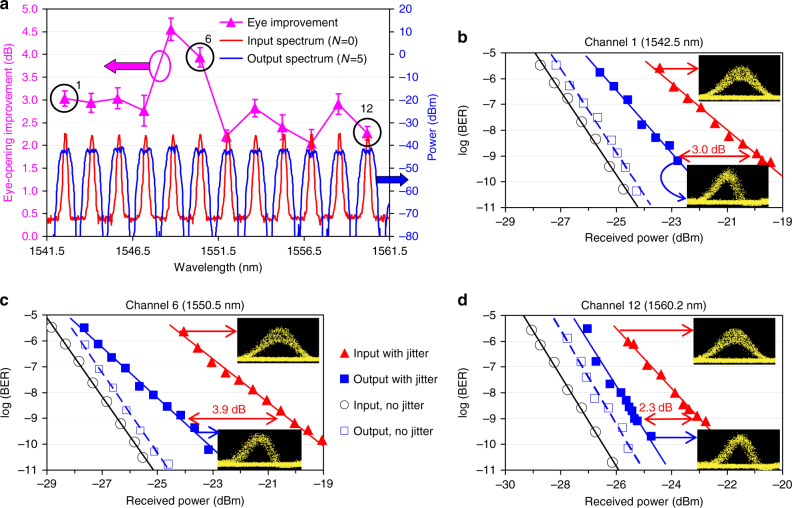
Results of 12-channel 2R regeneration in the recirculating loop. **a** Eye-opening improvements at BER = 10^−9^ for 12 simultaneously regenerated channels (*left axis*); 12-channel spectra at the input (*blue*) and output (*red*) of the regenerator (*right axis*), obtained with the resolution bandwidth of 0.1 nm. *N* is the number of circulations. *Error bars* are obtained from the linear fits to BER curves. *Numbered circles* indicate the channels shown in **b**–**d**. **b**–**d** BER curves and eye diagrams for amplitude-jitter-degraded signals before (*red triangles*) and after the regeneration (*blue squares*) for 1542.5-nm (**b**), 1550.5-nm (**c**) and 1560.2-nm (**d**) channels. BERs for signals without amplitude jitter are also shown (*black open circles*—before and *blue open squares*—after the regeneration). **b**–**d** Share common legend. The eye diagrams (insets in **b**–**d**) exhibit significant reduction in the amplitude jitter after the regeneration

The most important results of the recirculating-loop experiment are presented in Fig. 4a–d, demonstrating 2R regeneration of 12 10-Gbps channels with 200-GHz spacing. The data in Fig. 4a, obtained from comparing the curves of BER versus receiver pre-amplifier’s input power (examples of which are given in Fig. 4b–d), clearly indicate that the regenerator improves eye opening by more than 2 dB for all 12 channels. The eye diagrams in the insets of Fig. 4b–d also show significant reduction of the amplitude jitter after the regeneration. The number of channels in this experiment has been limited by the gain-flattened bandwidth of our high-power erbium-doped fibre amplifier (EDFA) (1542–1560 nm) and the 200-GHz channel spacing of the PGDD. The role of gain flattening is evident in Fig. 4a by the better performance of channels 5 and 6, for which the EDFA gain ripple is minimal. This results in the smallest input-to-output power excursion, a more even distribution of the SPM generation among all circulations, and nearly ideal regeneration conditions for these two channels.

### Demonstration of a stand-alone multi-channel 2R regenerator

**Fig. 5:**
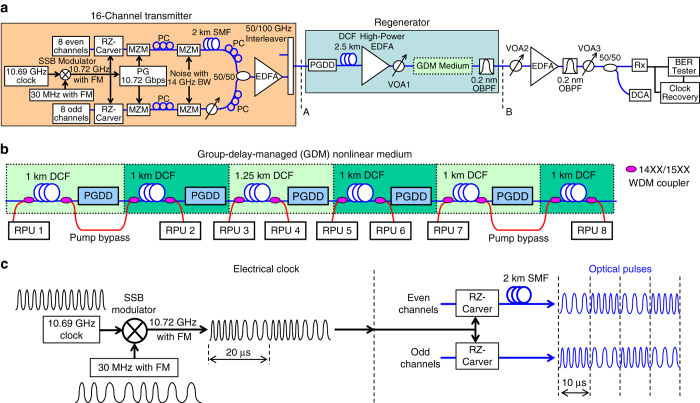
Stand-alone regenerator setup. **a** 10-Gbps-modulated signals from a 16-channel transmitter enter the regenerator, and their performance is characterised by a pre-amplified receiver. **b** GDM medium in the stand-alone regenerator consists of six Raman-pumped sections of DCF, separated by five PGDDs. PG: pattern generator, SSB: single sideband, SMF: single-mode fibre, RPU: Raman pump unit. **c** Decorrelation of the even- and odd-channel clock frequencies by frequency modulation (FM) of a single clock source

With the insights gained from the recirculating-loop experiments, we have built a stand-alone 2R regenerator (Fig. 5a) consisting of five PGDDs and six sections of nonlinear fibre (Fig. 5b), described in greater detail in ‘Methods’. Although an ideal PGDD is a pure all-pass filter and there are no fundamental limits on making it virtually lossless, the PGDDs that are commercially available today have finite insertion losses of 3–5 dB, primarily owing to the lack of market incentive to further reduce the loss in their current application (dispersion compensation at the loss-tolerant mid-stage of an EDFA). To compensate for the loss of the PGDDs, nonlinear fibre, couplers, and splices, and to achieve similar amounts of SPM in all fibre sections, we use bi-directional distributed Raman amplification in all sections of the nonlinear fibre. The number of regenerated WDM channels in our experiment is limited by the available Raman pump wavelengths and powers, which determine the spectral shape and magnitude of the saturated Raman gain. In order to avoid confusing 2R regeneration with artifacts of Raman gain saturation (which can suppress low-frequency amplitude jitter), we introduce fast amplitude fluctuations by modulating all channels with broadband (14-GHz-wide) Nyquist–Johnson electronic noise. The multi-channel regeneration is characterised by comparing the eye diagrams and ʽBER versus receiver input powerʼ curves between the regenerator input (point A in Fig. 5a) and regenerator output (point B in Fig. 5a) for all channels.

**Fig. 6:**
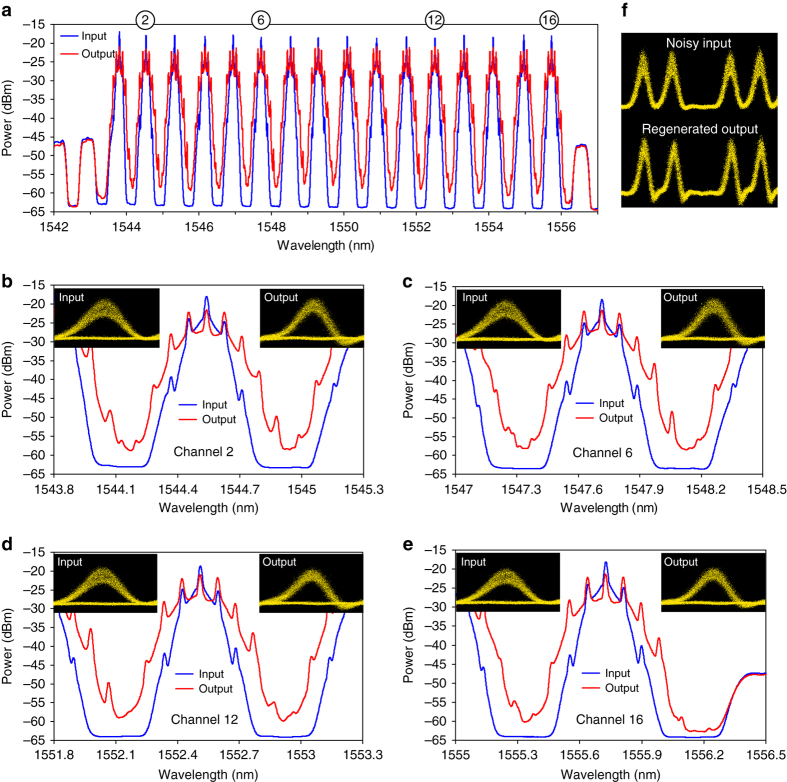
Spectra and eye-diagrams before and after the stand-alone 2R regenerator. **a**–**e** Optical spectra of the noise-degraded signals at the regenerator input (*blue)* and output (*red*) for all 16 channels (**a**) and four representative channels (detail view, **b**–**e**), obtained with the resolution bandwidth of 0.016 nm. Corresponding eye diagrams are shown as insets. Encircled numbers at the top of **a** show positions of the four channels. **f** 5-bit pattern before and after regeneration (1555.75-nm channel)

**Fig. 7:**
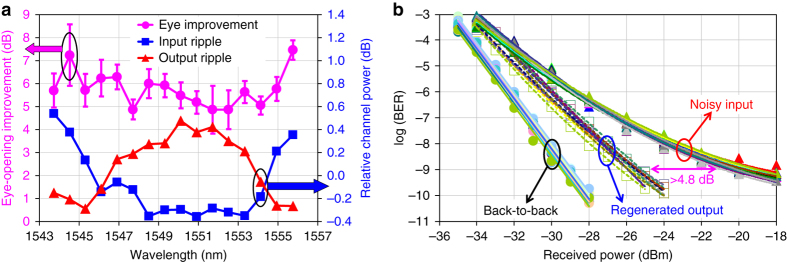
BER measurements and spectral ripple before and after the stand-alone 2R regenerator. **a** Eye-opening improvements at BER = 10^−9^ level (*purple circles* pertaining to the left axis) and spectral ripple (*blue squares* and *red triangles* pertaining to the right axis) among the 16 simultaneously regenerated channels. By pre-emphasising the channel powers so that the ripple of the WDM spectrum at the GDM medium input (*blue squares*) is the inverse of that at the GDM medium output (*red triangles*), we minimise the differences in optical signal-to-noise ratios and nonlinear phase shifts among the 16 channels due to Raman gain ripple^66^ *. Error bars* are obtained from the parabolic fits to BER curves. **b** BER curves for all 16 channels

The experimental results shown in Figs. 6 and 7 indicate similar amounts of the SPM-induced spectral broadening and similar eye-opening improvements for all 16 regenerated channels. In addition to presenting 16-channel spectra in Fig. 6a, we show detailed view of the spectra of four representative channels (channels 2, 6, 12 and 16) in Fig. 6b–e to illustrate that they indeed have similar shapes and similar amounts of nonlinear spectral broadening. The eye diagrams of these channels (insets in Fig. 6b–e) also exhibit similar and clearly noticeable amounts of the amplitude noise suppression by the regenerator. Figures 7a, b indicate that the eye-opening improvements for all 16 channels are 4.8 dB or better. Building upon the 12-channel recirculating-loop results, this 16-channel experiment represents the first, to the best of our knowledge, demonstration of a truly multi-channel 2R regenerating device. Moreover, it uses 100-GHz WDM channel spacing, which, as of the manuscript’s submission time, is the narrowest channel spacing among any 2R regenerators processing more than one WDM channel.

We emphasise that the number of channels in this experiment is not constrained by any conceptual limitations of the GDM approach, and is only limited by the bandwidth of the practical Raman amplifiers available in our lab. Hence, we expect this approach to be scalable to much higher channel counts when wider-bandwidth amplifiers are used.

## Discussion

There are two main reasons why the measured eye-opening improvements differ between the recirculating-loop and stand-alone regenerator experiments: (a) extra loss per unit cell (i.e. per circulation) inherent to operation of the recirculating loop and (b) different types of signal degradation used in the two experiments (see ‘Methods’ for more details), with broadband noise used in the stand-alone regenerator experiment having stronger impact on the BER. We would also like to note that the broadband noise is not only the most challenging, but also the most important impairment to regenerate. Indeed, the linear-optical transmission-line impairments (e.g. chromatic dispersion) can be easily overcome by simple multi-channel-compatible means, and many nonlinear-optical impairments (e.g. nonlinear inter-channel crosstalk) can be compensated in the near future either by digital back-propagation techniques^43^ or by multi-channel-compatible mid-span spectral inversion^44–46^. On the other hand, the distributed nonlinear interaction of the signal with the broadband noise has been shown to be the most fundamental capacity-limiting transmission-line impairment that can only be undone by regeneration^47, 48^. It is also worth noting that, although our artificial degradations in both recirculating-loop and stand-alone regeneration experiments primarily affect the amplitude of the ʽONESʼ symbols, the ʽZEROʼ symbols still carry the usual impairments inherent to transmission systems, such as finite ( ~ 13 dB) extinction ratio of the modulators, accumulation of the amplified spontaneous emission noise, etc. Thus, even though the ʽZEROʼ symbols are not artificially degraded, and their noise in the eye-diagram pictures is partially obscured by the receiver noise of the digital communication analyser (DCA), the measured BERs and eye-opening improvements represent the net performance contributed by both ʽONESʼ and ʽZEROSʼ. The ability of a GDM-based Mamyshev regenerator to improve the signal having strongly artificially degraded ʽZEROSʼ was previously demonstrated for a single channel^41^.

**Fig. 8:**
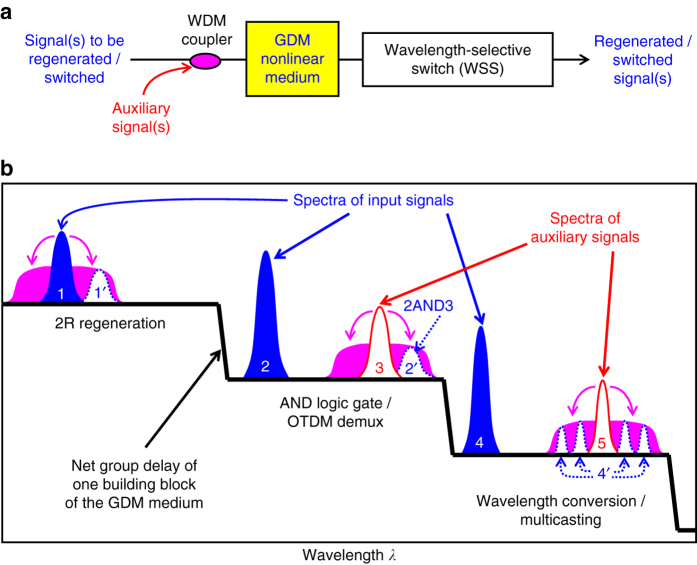
Schematics of different GDM-enabled all-optical processing applications. These different applications could be potentially implemented simultaneously and independently for signals at multiple wavelengths. **a** Multi-channel signals, combined with auxiliary beams, enter the GDM medium, after which the appropriate output beams are selected by a WSS. **b** Top step of the ʽstaircaseʼ in the group-delay spectra (*black*): Mamyshev 2R regenerator implemented for channel 1. Middle step: XPM-based AND operation (or OTDM demultiplexing) between channel 2 and auxiliary signal 3. Bottom step: wavelength conversion/multicasting of channel 4 by its XPM of auxiliary signal 5. Beams 1′, 2′ and 4′ (*dotted blue*) are selected by the WSS at the output, and the rest of the signals are discarded. Note that the GDM medium permits independent processing of multiple channels for as long as different signal channels (*solid blue*), along with their respective auxiliary beams (*red*), occupy different stairsteps of the group-delay staircase spectra

By employing lower-loss PGDDs and wider-bandwidth optical amplifiers while keeping the same number of unit cells, we expect the GDM-based approach to straightforwardly extend to even higher channel counts typical for WDM communication systems. Operation at higher symbol rates is also possible after proper rescaling of the signal powers and GDM parameters^21^ and has been numerically validated at 40 Gbps^21^. Although, as a proof of concept of GDM-based multi-channel regeneration, the presented experiments have only focused on the Mamyshev regenerator of OOK signals, we believe there is a clear path to extending this GDM approach to more advanced modulation formats in the future. In particular, the GDM nonlinear medium has been predicted to enable a multi-channel phase-preserving amplitude regeneration of phase-encoded signals^49, 50^ in a nonlinear amplifying loop mirror (NALM)^51, 52^, which is also capable of regeneration of multiple amplitude levels^53^. The GDM nonlinear medium easily permits bi-directional use, which is essential for NALM operation. Phase-preserving amplitude regenerators could further be paired^54, 55^ with potentially multi-channel (see, e.g. refs. ^56–60^) phase regenerators based on phase-sensitive parametric amplifiers^61–64^ to provide multi-channel processing of signals encoded with advanced modulation formats, leading to the capacity and reach increase for communication systems^65^. Such an extension of the GDM-assisted multi-channel regeneration to advanced modulation formats presents many challenges, but appears to be a promising direction for future research efforts. Another area for future investigation is in exploring the applications of the GDM nonlinear medium in other all-optical signal-processing schemes, where it may enable multi-channel operation by effectively decoupling the nonlinear interactions taking place within the channel bandwidth of the PGDD from the inter-channel interactions. We show one example of such a potential multi-channel processing scheme in Fig. 8.

## Methods

### Recirculating-loop experiment

The recirculating-loop setup uses a single ʽfibre + PGDDʼ unit cell with 1.25 km of DCF and ≈ + 20 ps nm^−1^ net cell dispersion^40^. Two sets of 400-GHz-spaced WDM channels are interleaved to form 12 200-GHz-spaced channels (1542.54…1560.20 nm) modulated by two independent 9.953-Gbps pattern generators (PGs) with slightly different and uncorrelated clock frequencies. We also add a 2-bit delay between the adjacent channels within each set, decorrelating their bit patterns originating from the same PG by a 0.5-km-long DCF. This ensures that we study a realistic worst-case scenario of co-polarised uncorrelated WDM channels, and average the performance over various initial delays between the pulses of neighbouring channels, as required for proper assessment of the inter-channel penalties in the regenerator^15, 21^. We degrade the channel selected for BER measurements by ±25% amplitude jitter via adding a crosstalk from another laser tuned to the measured channel’s wavelength. The remaining WDM channels are not artificially degraded. Each channel’s average power is  ~ 60 mW at the DCF input. An attenuator after the DCF prevents nonlinear interactions outside the DCF. The signals are injected into the loop by acousto-optic switch AOS1. AOS1 and AOS2 are driven by trains of rectangular pulses in counter-phase: during the loop-loading cycle, AOS1 is in a low-loss state and AOS2 is in a high-loss state; their states are reversed for the rest of the rectangular-wave period (loop recirculation). The net loss and gain in the loop are carefully balanced. Pulsed gate coinciding with timing of a particular circulation in the loop controls the inputs to the optical spectrum analyser (OSA), BER tester and DCA. After five circulations, the regenerated signals are selected, one at a time, by a 0.2-nm-wide tunable OBPF with  ~ 0.15-nm offset from the original signal’s wavelength and are subsequently detected by an optically pre-amplified receiver. It is worth noting that recirculating loops are most frequently used to characterise cascadability in optical networking and ultra-long-haul transmission, which typically requires 50 or more km of fibre (i.e. round-trip time at least in hundreds of μs) for reliable operation. In contrast, in our experiment, by using fast ( < 50 ns response time) loop switches and diagnostic equipment gates (acousto-optic switches AOS3 and AOS4 in front of the OSA and DCA, respectively, in Fig. 2), as well as an agile clock recovery at the receiver, we have adapted the recirculating loop to work with short round-trip times down to few μs (several hundred metres of fibre). This experiment used a fibre-Bragg-grating-based PGDD^30^ (TeraXion ClearSpectrum).

### Stand-alone-regenerator experiment

In the stand-alone-regenerator experiment, we use 16 WDM channels (1543.73…1555.75 nm) on a 100-GHz-spaced ITU grid, obtained by combining even and odd 8-channel sets. Since all signals are modulated by the same 10.7-Gbps PRBS, we decorrelate the bit patterns between the even and odd WDM channel sets by 2-km-long single-mode fibre (SMF) in the even-channel path. We also decorrelate the bit patterns within each of the even and odd channel sets by sending the combined signals through 2.5 km of DCF, producing a 5-bit pattern delay between the adjacent channels of each 200-GHz-spaced set. The intra-channel dispersion from this DCF spool is 67%-compensated by a preceding PGDD (set to + 200 ps nm^−1^), while the dispersion accumulated between the channels is preserved. To emulate the real noise in transmission, a broadband Nyquist–Johnson thermal noise, generated by several cascaded electronic amplifiers with net frequency range spanning from 50 kHz to 14 GHz, drives separate amplitude modulators (of the same type as those used for data modulation) for even and odd channels and is decorrelated among the channels by the same combination of the 2-km-long SMF and 2.5-km-long DCF. In addition, we decorrelate the clock frequencies of even and odd channels by frequency modulation (FM) of the clock of a single PG, in lieu of employing a second PG. This is done by mixing the original 10.69 GHz sine-wave clock at a radio-frequency (RF) single-sideband modulator with a 30-MHz sine wave, FM-modulated with 50-kHz repetition frequency and  ± 100-kHz frequency deviation, which generates a 10.72-GHz clock with FM, as shown in Fig. 5c. The half-period of FM (10 μs) roughly corresponds to the delay of the even channels by the 2-km-long SMF and ensures that, after combining, even and odd channels have clocks whose frequency difference constantly changes in the range from –200 to +200 kHz, leading to continuous variation of the relative input bit delays between the adjacent 100-GHz-spaced channels from –0.64 to +0.64 bit periods over the span of 20-μs FM period. After the first EDFA, a 50-to-100-GHz interleaver with 0.25-nm-wide passband suppresses out-of-band amplified spontaneous emission noise. After the second, high-power, EDFA the co-polarised signals enter the Mamyshev regenerator consisting of the GDM medium producing SPM-induced spectral broadening and an OBPF detuned by –0.07 nm from the original channel’s centre. In a real system, a spectrally periodic OBPF (comb filter such as an interleaver) can simultaneously handle all WDM channels, whereas we use a single tunable 0.2-nm-wide OBPF, because we only have one receiver. The GDM medium consists of five identical 1-km-long DCF sections and one 1.25-km DCF section near the middle of the GDM medium, all separated by PGDDs. This experiment uses PGDDs based on cascaded Gires–Tournois etalons (Oclaro PowerShaper PS3300), which have flat amplitude response^29^. The dispersion of each PGDD is set to +150 ps nm^−1^, yielding the residual dispersion for each of four identical (1-km DCF + PGDD) unit cells near +30 ps nm^−1^ optimum^40^ and the residual dispersion of the cell with 1.25-km-long DCF near 0 ps nm^−1^. Compared to our preliminary experiments^26^, the residual dispersion of each cell has been increased by 20 ps nm^−1^ and the pre-compensation (DCF + PGDD preceding the GDM medium) has been changed from 0 to –100 ps nm^−1^ to produce better regeneration performance. The net intra-channel dispersion of the entire GDM medium including the last piece of 1-km-long DCF is 0 ps nm^−1^. At the same time, the signals from the adjacent 100-GHz-spaced WDM channels walk off from each other by at least 6 bit periods over the GDM medium length. Each PGDD’s loss is  ≈ 4 dB, which, together with the loss of the DCF (including DCF-to-SMF splices) and pump/signal WDM couplers, yields the total passive loss of the GDM medium near 35 dB. To compensate it, we use eight Raman pump units (RPUs, each with two wavelengths and two polarisations multiplexed) to bi-directionally pump the GDM medium to transparency (with undepleted pumps). RPU #1 in Fig. 5b has pump wavelengths of 1440 nm and 1455 nm, RPU #2—1443 nm and 1455 nm, RPU#3–#6—1460 nm and 1470 nm, RPU#7—1440 nm and 1462 nm, and RPU#8—1443 and 1462 nm. Extra pump/signal 14XX/15XX WDM couplers allow the Raman pump light bypass the lossy PGDDs. The optimum total average signal power entering the GDM medium is 26 dBm (~ 25 mW per channel). At this power the Raman gain is saturated, and the total average signal power at the output of the last 1-km DCF section falls to 23.6 dBm. Compared to our preliminary experiments^26^, in this setup we have shifted the WDM channel plan toward shorter wavelengths to take advantage of the Raman gain peak of the available pumps, and optimised the Raman pump powers to better equalise the gain. This has increased the minimum (worst-channel) GDM net transmission from –5.6 dB to –3.2 dB and reduced the gain ripple from 2.8 dB to 1.4 dB, yielding considerably less variation of the signal power across the spectrum and along the length of the GDM medium, i.e. better approximating the ideal lossless GDM medium. As a result, we have achieved a 33% increase in the number of WDM channels (from 12 to 16), as well as a better and more uniform regeneration performance among the WDM channels.

### Data availability

The data that support the findings of this study are available from the corresponding author upon reasonable request.
